# Effect of Diosgenin in Suppressing Viability and Promoting Apoptosis of Human Prostate Cancer Cells: An Interplay with the G Protein-Coupled Oestrogen Receptor?

**DOI:** 10.3390/ijms252212006

**Published:** 2024-11-08

**Authors:** Marília I. Figueira, Ricardo Marques, Henrique J. Cardoso, Lara R. S. Fonseca, Ana P. Duarte, Samuel Silvestre, Sílvia Socorro

**Affiliations:** 1 CICS-UBI—Health Sciences Research Centre, University of Beira Interior, 6201-001 Covilhã, Portugal; marilia.figueira@fcsaude.ubi.pt (M.I.F.); henrique10mc@gmail.com (H.J.C.); lara@fcsaude.ubi.pt (L.R.S.F.); apcd@ubi.pt (A.P.D.); sms@ubi.pt (S.S.); 2 Instituto Politécnico da Guarda (IPG), 6300-559 Guarda, Portugal; rmarkes14129@hotmail.com

**Keywords:** diosgenin, GPER, prostate cancer, cell viability, apoptosis, metabolism

## Abstract

Diosgenin is a phytosteroid sapogenin with reported antitumoral activity. Despite the evidence indicating a lower incidence of prostate cancer (PCa) associated with a higher consumption of phytosteroids and the beneficial role of these compounds, only a few studies have investigated the effects of diosgenin in PCa, and its mechanisms of action remain to be disclosed. The present study investigated the effect of diosgenin in modulating PCa cell fate and glycolytic metabolism and explored its potential interplay with G protein-coupled oestrogen receptor (GPER). Non-neoplastic (PNT1A) and neoplastic (LNCaP, DU145, and PC3) human prostate cell lines were stimulated with diosgenin in the presence or absence of the GPER agonist G1 and upon GPER knockdown. Diosgenin decreased the cell viability, as indicated by the MTT assay results, which also demonstrated that castrate-resistant PCa cells were the most sensitive to treatment (PC3 > DU145 > LNCaP > PNT1A; IC50 values of 14.02, 23.21, 56.12, and 66.10 µM, respectively). Apoptosis was enhanced in diosgenin-treated cells, based on the increased caspase-3-like activity, underpinned by the altered expression of apoptosis regulators evaluated by Western blot analysis, which indicated the activation of the extrinsic pathway. Exposure to diosgenin also altered glucose metabolism. Overall, the effects of diosgenin were potentiated in the presence of G1. Moreover, diosgenin treatment augmented GPER expression, and the knockdown of the *GPER* gene suppressed the proapoptotic effects of diosgenin in PC3 cells. Our results support the antitumorigenic role of diosgenin and its interest in PCa therapy, alone or in combination with G1, mainly targeting the more aggressive stages of the disease.

## 1. Introduction

Prostate cancer (PCa) is an endocrine-related cancer highly dependent on steroid hormone actions [1,2,3,4,5,6,7,8,9]. The hormone-sensitivity of PCa, namely to androgens and oestrogens (i.e., being stimulated by androgenic activity and inhibited by suppression of androgen levels or oestrogen administration) has been the basis for using hormone therapies in PCa treatment [10,11]. However, the management of PCa faces limitations due to the associated adverse side effects and development of resistance mechanisms. The emergence of castration-resistant PCa (CRPC) remains a major clinical challenge as it is a stage of the disease where androgen-deprivation therapies become ineffective, leading to limited therapeutic options and poor prognosis for patients [12], highlighting the need for novel and targeted approaches in PCa treatment.

The lower incidence of PCa in Eastern countries, associated with a plant-based diet, the higher consumption of phytosteroids [13,14,15,16], and the garnered attention to the anti-cancer properties of these natural compounds [17,18], have stimulated research exploring their actions in PCa.

Diosgenin, (25R)-Spirost-5-en-3β-ol, is a phytosteroid sapogenin found mainly in the tubers and seeds of some species of Dioscorea, Costus, Smilax, and Trigonella [19,20,21,22]. This biologically active steroidal sapogenin has a structure similar to cholesterol and other steroids and has been used by the pharmaceutical industry as a starting material in the synthesis of steroid hormones and other drugs (623, 624). In addition, diosgenin has attracted significant interest as a therapeutic agent, particularly due to its ability to modulate key cellular processes in cancer development and progression [23,24]. In fact, previous studies have indicated that diosgenin can reduce tumour development and progression [25,26,27]. However, the precise mechanisms by which diosgenin exerts anticancer effects, particularly in PCa cells, remain to be fully elucidated. Significant gaps also exist considering the effect of diosgenin over the different cancer hallmarks, namely the metabolic reprogramming typical of cancer cells, characterised by enhanced glycolytic activity with the production of lactate to the detriment of oxidative phosphorylation, even in aerobic conditions [18,28,29,30].

The G protein-coupled oestrogen receptor (GPER), a membrane-bound receptor distinct from the classical nuclear oestrogen receptors (ERα and ERβ), has been implicated in the regulation of PCa cell growth, and its activity is thought to intersect with various oncogenic signalling pathways [31,32,33,34,35,36,37,38,39,40,41,42,43,44,45,46]. Emerging evidence indicates that diosgenin could act by activating ERα and ERβ [47,48,49], but nothing is known about its interaction with GPER. Nevertheless, it has been reported that other phytosteroids could activate GPER [50,51,52,53,54,55,56,57], raising curiosity about the role of GPER-mediated signalling in diosgenin actions.

This study aimed to investigate the effect of diosgenin in modulating PCa cell fate and glycolytic metabolism and explore the potential interplay with GPER signalling. For this purpose, experiments with small interfering RNA (siRNA) GPER knockdown and GPER agonist G1 [58] were performed. G1 (GPR30-specific compound 1) is a selective agonist for the GPER, which has garnered attention in a diversity of studies due to the demonstration of oestrogen signalling triggered at the cell membrane and mediated by GPER. Its actions in different physiological effects and conditions including cancer have been shown [31,59].

## 2. Results

### 2.1. Diosgenin Treatment Reduced the Viability of PCa Cells

**Figure 1:**
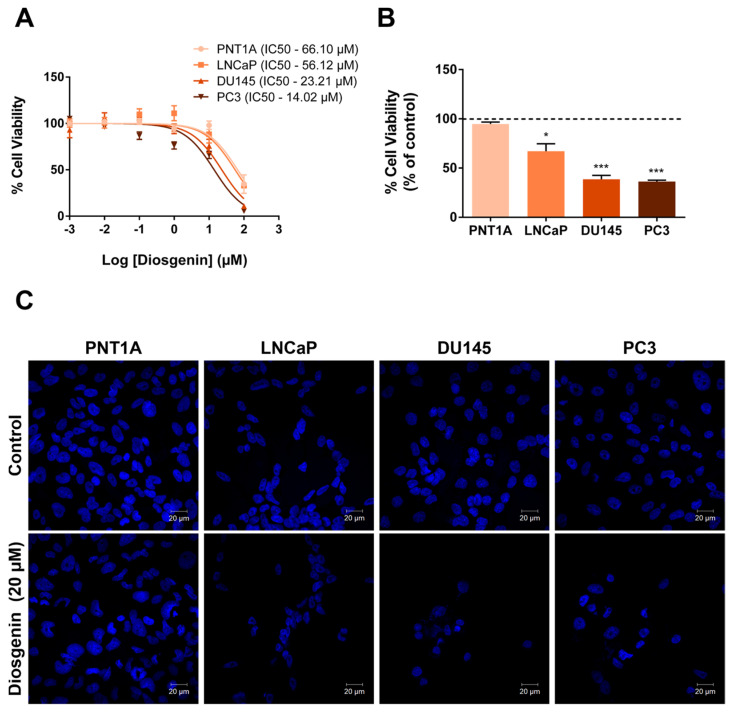
Effect of diosgenin on the viability of non-neoplastic (PNT1A) and neoplastic (LNCaP, DU145, and PC3) human prostate cells. (**A**) Diosgenin concentration-–response curve and IC50 determined by the MTT analysis of cell viability with a concentration range from 0.001 to 100 µM for 24 h. The IC50 value was obtained by the logarithmic transformation of the concentrations. (**B**) Cell viability after treatment with 20 µM diosgenin for 24 h. For each cell line, results are expressed as % of control (0 µM diosgenin, dashed line). Error bars indicate mean S.E.M. (n = 5). * *p* < 0.05, *** *p* < 0.001. (**C**) Representative fluorescence microscopy images of Hoechst 33342 stained cell nuclei in the control and diosgenin-treated cells. Images were acquired using a Zeiss LSM 710 laser scanning confocal microscope (Carl Zeiss, Göttingen, Germany) under 400× magnification.

To investigate the effect of diosgenin in human prostate cells, we started by exposing PNT1A, LNCaP, DU145, and PC3 cells to a concentration range of diosgenin (0.001 to 100 µM) for 24 h, and the cell viability was analysed (Figure 1). The highest diosgenin concentration (100 µM) markedly diminished the viability of all prostate cell line models (Figure 1A). Moreover, in the DU145 and PC3 cells, for concentrations higher than 0.1 µM, the diosgenin effects were concentration-dependent (Figure 1A).

The non-neoplastic PNT1A cells were shown to be less sensitive to diosgenin, with an IC50 of 66.10 µM (Figure 1A). The IC50 concentration in LNCaP cells was around 56.12 µM, whereas in the CRPC cells DU145 and PC3, lower IC50 values of 23.21 and 14.02 µM, were determined, respectively (Figure 1A).

The concentration of diosgenin selected for use in the subsequent experiments was 20 µM, as it reduced the DU145 and PC3 cell viability by approximately 50% and represents an intermediate IC50 value considering the results in all prostate cell lines. In a separate experiment, it was confirmed that 20 µM diosgenin did not affect the viability of PNT1A cells (Figure 1B) while significantly diminishing the viability of PCa cells compared to the respective control groups (approximately 30% reduction in LNCaP cells and 60% in DU145 and PC3, Figure 1B). Representative images of Hoechst-stained nuclei (Figure 1C) support the decrease in the viability of the PCa cells and cell number upon exposure to 20 µM diosgenin, which was visible compared with the unaltered cell number/viability in the PNT1A cells (Figure 1C).

### 2.2. Diosgenin Effects in Suppressing the Viability of PCa Cells Are Increased in the Presence of the GPER Agonist G1

**Figure 2:**
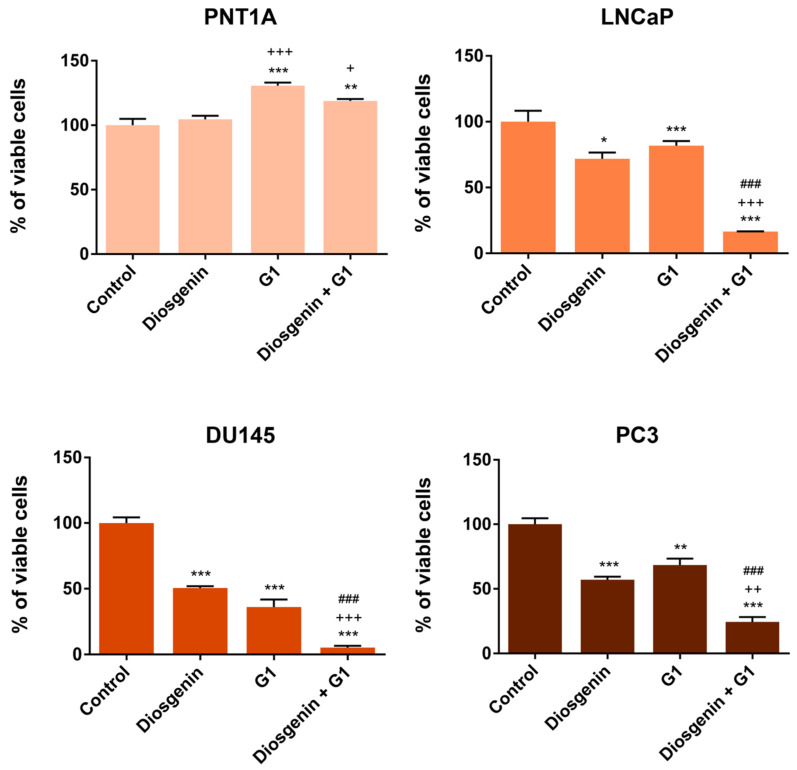
Viability of non-neoplastic (PNT1A) and neoplastic (LNCaP, DU145, and PC3) human prostate cells in response to diosgenin and GPER agonist G1. Cells were treated with 20 µM diosgenin and/or 1 µM G1 for 24 h and viability was determined by the MTT assay. Results are expressed as % of control. Error bars indicate mean ± S.E.M. (n = 3). * *p* < 0.05, ** *p* < 0.01, *** *p* < 0.001 compared to control; + *p* < 0.05, ++ *p* < 0.01, +++ *p* < 0.001 compared to diosgenin; ### *p* < 0.001 compared to G1.

PNT1A, LNCaP, DU145, and PC3 cells were treated with 20 µM diosgenin in the presence or absence of 1 µM GPER agonist G1. As expected, diosgenin did not influence PNT1A cell viability while it suppressed the viability of LNCaP, DU145, and PC3 cells by approximately 28, 49, and 43%, respectively (Figure 2). G1 alone also reduced the LNCaP, DU145, and PC3 cell viability by approximately 18, 64, and 32%, respectively (Figure 2). However, it increased the viability of the PNT1A cells (~31%). The combined treatment of diosgenin plus G1 had a highly pronounced effect, significantly diminishing the PCa cell viability, which was reduced by 95, 83, and 76% in the DU145, LNCaP, and PC3 cells, respectively. Diosgenin did not change the effect of G1 increasing the viability of the PNT1A cells (Figure 2).

**Figure 3:**
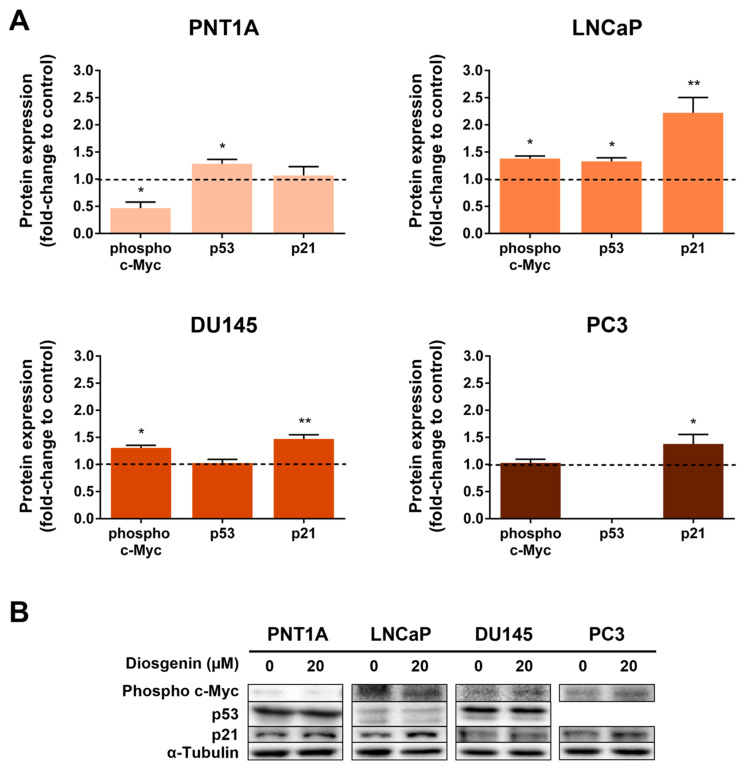
Effect of diosgenin on the expression of cell cycle regulators in non-neoplastic (PNT1A) and neoplastic (LNCaP, DU145, and PC3) human prostate cells. Cells were treated with 20 µM diosgenin for 24 h. (**A**) Protein expression of phospho c-Myc, p53, and p21 determined by WB analysis after normalisation with α-tubulin. Results are expressed as fold-change relative to the control untreated group (0 µM diosgenin, dashed line). Error bars indicate mean ± S.E.M. (n = 5). * *p* < 0.05, ** *p* < 0.01. (**B**) Representative immunoblots.

Considering the effects of diosgenin in suppressing PCa cell viability, we evaluated its influence on the expression of cell cycle regulators, namely phospho c-Myc, p53, and p21 (Figure 3) as they are target molecular players implicated in PCa [60]. Diosgenin treatment reduced the expression of phospho-c-Myc (0.47 ± 0.11-fold-change relative to control) in the PNT1A cells (Figure 3A). However, the phospho-c-Myc expression levels increased in the PCa cells. Exposure to diosgenin increased phospho-c-Myc expression in the LNCaP and DU145 cells by 1.38 ± 0.05 and 1.31 ± 0.05-fold-change relative to the control, respectively (Figure 3A,B). No changes were observed in phospho-c-Myc in PC3-treated cells (Figure 3A,B).

PNT1A and LNCaP showed increased p53 expression after treatment with diosgenin (1.28 ± 0.08 and 1.33 ± 0.07-fold-change relative to the control, respectively, Figure 3A). p53 was not detected in the PC3 cells, as these cells are known to be p53 negative [61]. Concerning p21, diosgenin increased its expression in all PCa cell lines (2.22 ± 0.28, 1.47 ± 0.08, and 1.38 ± 0.17-fold-change relative to the control in LNCaP, DU145, and PC3, respectively, Figure 3A,B). Diosgenin did not change the p21 expression in PNT1A cells (Figure 3A,B).

### 2.3. Diosgenin Increased GPER Expression in Non-Neoplastic and Neoplastic Human Prostate Cells

**Figure 4:**
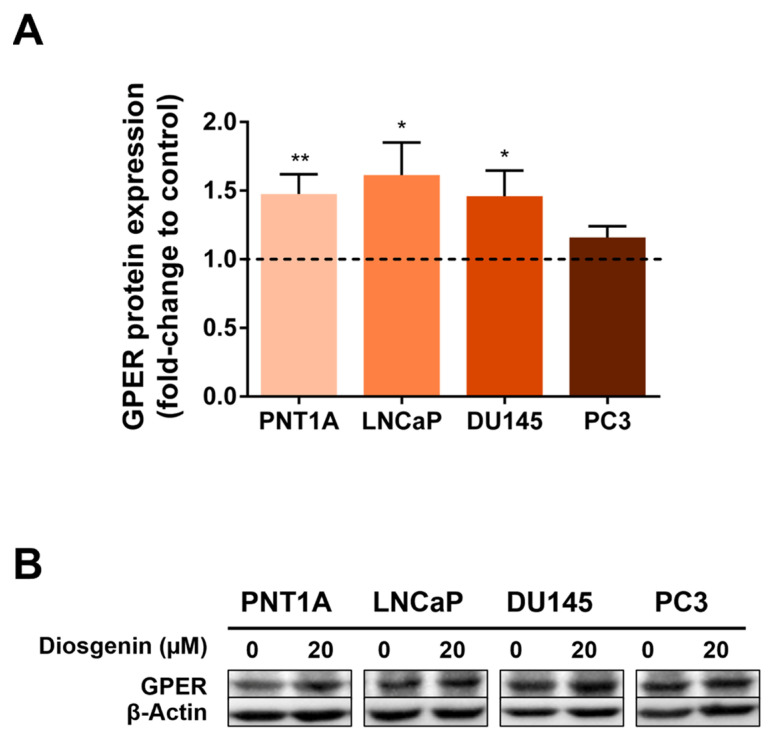
Effect of diosgenin in regulating GPER expression in non-neoplastic (PNT1A) and neoplastic (LNCaP, DU145, and PC3) human prostate cells. Cells were treated with 20 µM diosgenin for 24 h. (**A**) GPER protein expression determined by WB analysis after being normalised with β-actin. Results are expressed as fold-change relative to the control untreated group (0 µM diosgenin, dashed line). Error bars indicate mean ± S.E.M. (n = 5). * *p* < 0.05, ** *p* < 0.01. (**B**) Representative immunoblots.

Previously published data indicated that some phytosteroids could reduce PCa growth by modulating GPER expression [18]. Therefore, we decided to investigate whether diosgenin regulates GPER expression in prostate cells. Non-neoplastic PNT1A and neoplastic LNCaP, DU145, and PC3 cells were treated with 20 µM diosgenin for 24 h, and GPER protein expression was analysed by Western blot (WB). Diosgenin treatment increased GPER expression in both the non-neoplastic PNT1A (1.48 ± 0.14-fold-change relative to control) and neoplastic LNCaP and DU145 cells (1.61 ± 0.24 and 1.46 ± 0.19-fold-change relative to control, respectively) (Figure 4A,B). No changes were observed in GPER expression in thee PC3 cells after diosgenin stimulation (Figure 4A,B).

### 2.4. Diosgenin-Induced Apoptosis of Prostate Cells Is Enhanced by G1 and Suppressed by GPER Knockdown

**Figure 5:**
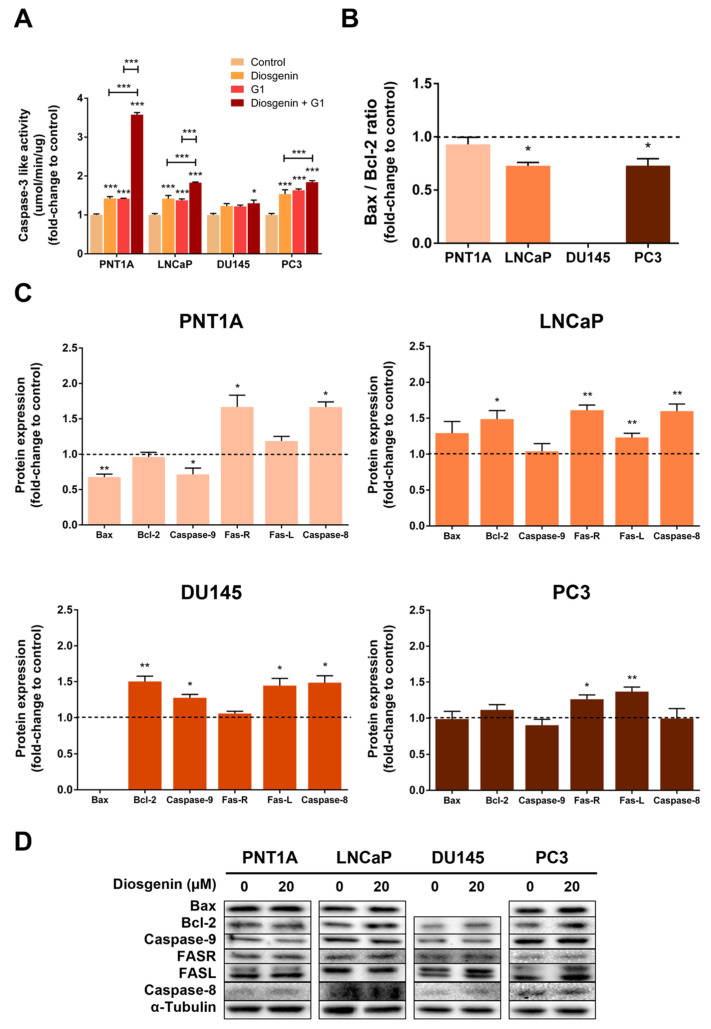
Effect of diosgenin and G1 in non-neoplastic (PNT1A) and neoplastic (LNCaP, DU145, and PC3) human prostate cell apoptosis. Cells were treated with 20 µM diosgenin and/or 1 µM G1 for 24 h. (**A**) Caspase-3-like activity determined spectrophotometrically. (**B**) Bax and Bcl-2 protein ratio. (**C**) Protein expression of regulators of the intrinsic and extrinsic pathways of apoptosis determined by WB analysis after normalisation with α-tubulin. Results are expressed as fold-change relative to the control group (0 µM diosgenin, dashed line). Error bars indicate mean ± S.E.M. (n = 5). * *p* < 0.05, ** *p* < 0.01, *** *p* < 0.001. (**D**) Representative immunoblots.

To evaluate the role of diosgenin in modulating prostate cell apoptosis, caspase-3-like activity was assessed in the PNT1A, LNCaP, DU145, and PC3 cells after treatment with diosgenin or/and G1. Caspase-3-like activity was augmented in the PNT1A, LNCaP, and PC3 cells (1.42 ± 0.05, 1.42 ± 0.08, and 1.54 ± 0.11-fold-change relative to control, respectively, Figure 5A) and no effect was observed in the DU145 cells upon exposure to diosgenin. Additionally, G1 increased caspase-3-like activity in all cell lines except for DU145 (1.42 ± 0.01, 1.38 ± 0.03, and 1.63 ± 0.03-fold-change relative to control in the PNT1A, LNCaP, and PC3 cells, respectively, Figure 5A).

The diosgenin effects were accentuated in the presence of G1. Caspase-3-like activity was significantly enhanced in the diosgenin plus G1-treated PNT1A (3.58 ± 0.05-fold-change relative to control), LNCaP (1.83 ± 0.02-fold-change relative to control), and PC3 (1.84 ± 0.04-fold-change relative to control) cells compared to the effect of diosgenin alone (Figure 5A). Furthermore, in contrast to the use of compounds alone, the combination of diosgenin plus G1 increased the caspase-3-like activity in the DU145 cells (1.30 ± 0.08-fold-change relative to the control, Figure 5A).

The expression of regulators of both the intrinsic and extrinsic pathways of apoptosis was also evaluated (Figure 5B–D). Diosgenin reduced the Bax/Bcl-2 ratio in the LNCaP and PC3 cells (0.73 ± 0.03 and 0.73 ± 0.06-fold-change relative to control, respectively, Figure 5B). In the PNT1A cells, despite the reduced expression of the pro-apoptotic protein Bax in response to diosgenin (0.68 ± 0.04-fold-change relative to the control, Figure 5C,D), the Bax/Bcl-2 ratio remained unchanged (Figure 5B). Concerning the PCa cells, diosgenin increased Bax expression in the LNCaP cells (1.74 ± 0.18-fold change to the control, Figure 5C,D). The Bax/Bcl-2 ratio was not calculated for DU145 because these cells do not express the Bax protein [62,63,64].

The expression levels of Bcl-2 (anti-apoptotic) increased after diosgenin treatment (Figure 5C,D) in the LNCaP and DU145 cells (1.49 ± 0.12 and 1.50 ± 0.07-fold-change relative to control, respectively, Figure 5C,D), with no effects being perceived in PNT1A and PC3.

Levels of the initiator caspase-9 upon exposure to diosgenin were increased in the DU145 cells only (1.28 ± 0.05-fold change to control, Figure 5C,D). In contrast, diosgenin significantly reduced caspase-9 expression in the PNT1A cells (0.72 ± 0.09-fold-change relative to the control, Figure 5C,D).

Regarding the extrinsic pathway of apoptosis, treatment with diosgenin increased FASR expression in the non-neoplastic cells PNT1A (1.67 ± 0.17-fold change to the control, Figure 5C,D) and in the LNCaP and PC3 cells (1.61 ± 0.07 and 1.26 ± 0.06-fold-change relative to the control, respectively, Figure 5C,D). Additionally, diosgenin induced FASL expression in the PCa cells (1.23 ± 0.06, 1.45 ± 0.10, and 1.37 ± 0.06-fold-change relative to the control for LNCaP, DU145, and PC3, respectively, Figure 5C,D). Caspase-8 expression was increased in response to diosgenin stimulation in the PNT1A, LNCaP, and DU145 cells (1.67 ± 0.07, 1.60 ± 0.10, and 1.48 ± 0.10-fold-change relative to the control, respectively, Figure 5C,D).

**Figure 6:**
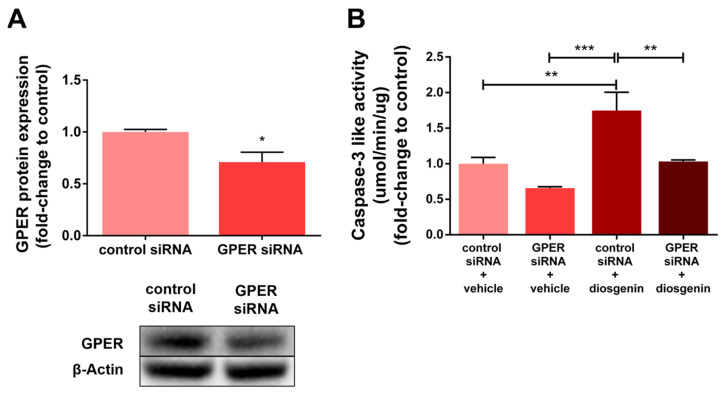
GPER knockdown abrogates the diosgenin effects inducing the apoptosis of human neoplastic PC3 cells. Cells were transfected with 40 nM of a nontargeting siRNA (control siRNA) or an siRNA targeting GPER (GPER siRNA) using lipofectamine RNAiMAX. (**A**) GPER protein expression determined by WB analysis after normalisation with β-actin. Representative immunoblots are shown in the bottom panel. (**B**) Caspase-3-like activity determined spectrophotometrically. Results are expressed as fold-change to the control siRNA + vehicle group. Error bars indicate mean ± S.E.M. (n = 3). * *p* < 0.05, ** *p* < 0.01, *** *p* < 0.001.

Considering the results obtained and to investigate whether diosgenin can act via GPER, PC3 cells were transfected with siRNA targeting GPER and scramble siRNAs (control) and the caspase-3-like activity was evaluated (Figure 6). GPER siRNA transfection significantly reduced the GPER protein expression by approximately 30% (Figure 6A). GPER knockdown suppressed the effect of diosgenin, increasing caspase-3-like activity in the PC3 cells (Figure 6B).

### 2.5. Diosgenin Modulated the Glycolytic Metabolism of Prostate Cells

**Figure 7:**
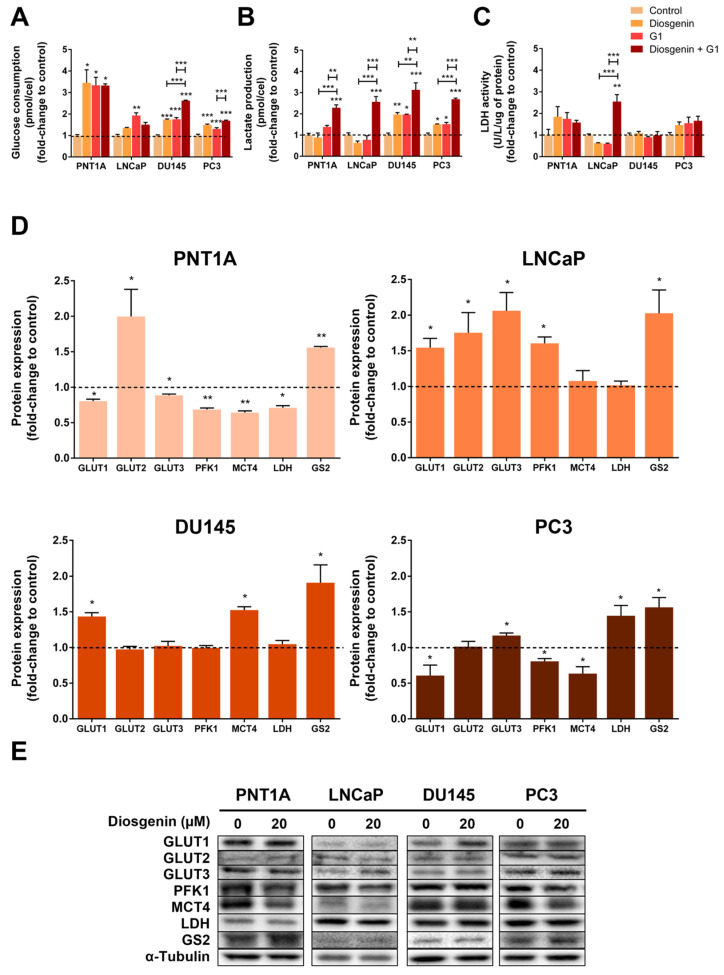
Diosgenin and G1 effects in modulating the glycolytic profile of non-neoplastic (PNT1A) and neoplastic (LNCaP, DU145, and PC3) human prostate cells. Cells were treated with 20 µM diosgenin and/or 1 µM G1 for 24 h. (**A**) Glucose consumption, (**B**) lactate production, and (**C**) LDH activity, determined spectrophotometrically. (**D**) Protein expression of glycolytic metabolism regulator proteins determined by WB analysis after normalisation with α-tubulin. Results are expressed as fold-change to the control untreated group (0 µM diosgenin and 0 µM G1, dashed line). Error bars indicate mean ± S.E.M. (n = 5). * *p* < 0.05, ** *p* < 0.01, *** *p* < 0.001. (**E**) Representative immunoblots.

Metabolic reprogramming with the switch from oxidative phosphorylation to glycolysis, even in aerobic conditions, is a well-known characteristic of cancer cells [65,66], which in the case of PCa is mainly associated with the advanced stages of disease [29,67,68]. These metabolic alterations were established as a cancer hallmark in the last decade as they highly sustain cancer cell growth and invasiveness [29,30]. Therefore, we aimed to investigate the effect of diosgenin on the glycolytic profile of prostate cells. Stimulation with diosgenin significantly increased the glucose consumption in the PNT1A and CRPC cells (3.45 ± 0.61, 1.74 ± 0.03, and 1.49 ± 0.04-fold-change relative to the control in the PNT1A, DU145, and PC3 cells, respectively, Figure 7A), with no significant effect in the LNCaP cells (Figure 7A). Additionally, G1 augmented glucose consumption in all cell lines (3.34 ± 0.37,1.93 ± 0.12, 1.76 ± 0.07, and 1.31 ± 0.06-fold-change relative to the control in the PNT1A, LNCaP, DU145, and PC3 cells, respectively, Figure 7A). Moreover, the combination of diosgenin and G1 was shown to improve the diosgenin and/or G1-induced augmentation of glucose consumption in the CRPC cells (2.62 ± 0.02 and 1.69 ± 0.03-fold-change relative to the control in the DU145 and PC3 cells, respectively, Figure 7A). In PNT1A, the combined diosgenin and G1 treatment did not affect glucose consumption compared to the compounds alone (Figure 7A).

The increased consumption of glucose in the diosgenin- or G1-treated CRPC cells was accompanied by the enhanced lactate production. Diosgenin increased the lactate production by 1.97 ± 0.09 and 1.50 ± 0.03-fold-change relative to the control in the DU145 and PC3 cells, respectively (Figure 7B). For G1, the variation was 1.97 ± 0.02 and 1.51 ± 0.09-fold-change relative to the control in the DU145 and PC3 cells, respectively (Figure 7B). Furthermore, the combined diosgenin and G1 treatment strongly increased the lactate production in all cell lines by approximately 2–3 times compared to the control group (Figure 7B). Nevertheless, the activity of lactate dehydrogenase (LDH), the enzyme responsible for the conversion of pyruvate to lactate, only changed in the diosgenin plus G1-treated LNCaP cells (approximately 2.5-fold-change compared to the control group, Figure 7C). LDH expression was reduced by diosgenin in the PNT1A cells (0.71 ± 0.03-fold-change relative to the control, Figure 7D,E). On the other hand, diosgenin increased the LDH protein levels in the PC3 cells (1.45 ± 0.14-fold-change relative to the control); no changes were perceived on the expression of LDH in the LNCaP and DU145 cells (Figure 7D,E).

Diosgenin reduced glucose transporter 1 (GLUT1) expression in the PNT1A and PC3 cells (0.80 ± 0.03 and 0.61 ± 0.15-fold-change relative to the control, respectively, Figure 7D,E), but the diosgenin-treated LNCaP and DU145 cells displayed augmented GLUT1 expression (1.54 ± 0.13 and 1.44 ± 0.05-fold-change relative to the control, respectively, Figure 7D,E).

GLUT2 expression was increased by diosgenin treatment in the non-neoplastic PNT1A cells (2.00 ± 0.38-fold-change relative to the control, Figure 7D,E) and in the LNCaP cells (1.75 ± 0.28-fold-change relative to the control), whereas no changes were observed in the DU145 or PC3 cells (Figure 7D,E).

Regarding GLUT3, diosgenin reduced its expression in PNT1A cells (0.80 ± 0.03 0.89 ± 0.02 and 0.88 ± 0.02-fold-change relative to the control, respectively Figure 7D,E) and augmented it in the LNCaP and PC3 cells (2.06 ± 0.26 and 1.17 ± 0.04-fold-change relative to the control, respectively, Figure 7D,E). No effect was observed in the DU145 cells (Figure 7D,E).

Phosphofructokinase 1 (PFK1) is an enzyme that catalyses a limiting step of glycolysis, converting fructose 6-phosphate to fructose 1,6-bisphosphate [69]. Stimulation with diosgenin reduced PFK1 expression in the PNT1A and PC3 cells (0.68 ± 0.02 and 0.81 ± 0.04-fold-change relative to the control, respectively, Figure 7D,E). On the other hand, diosgenin increased PFK1 expression in the LNCaP cells (1.60 ± 0.09-fold-change relative to the control, Figure 7D,E). No changes were observed in PFK1 expression in the diosgenin-treated DU145 cells (Figure 7D,E).

The expression of monocarboxylate transporter 4 (MCT4), the MCT responsible for lactate export, was reduced by diosgenin in the PNT1A (0.64 ± 0.03-fold-change relative to control) and PC3 cells (0.63 ± 0.10-fold-change relative to the control, Figure 7D,E). In contrast, DU145 displayed increased expression of MCT4 after treatment with diosgenin (1.52 ± 0.05-fold-change relative to the control); no changes were observed in the LNCaP cells (Figure 7D,E). Diosgenin was also found to augment the glycogen synthase 2 (GS2) expression levels in all cell lines (PNT1A: 1.56 ± 0.02; LNCaP: 2.03 ± 0.33; DU145: 1.91 ± 0.25; and PC3: 1.56 ± 0.14-fold-change relative to the control, Figure 7D,E).

## 3. Discussion

The present work investigated the effects of diosgenin and the contribution of this natural compound in counteracting PCa cell features. We started by characterising the concentration–response curves of non-neoplastic (PNT1A) and neoplastic (LNCaP, DU145, and PC3) human prostate cells to diosgenin. The prostate cell viability was reduced in a concentration-dependent manner, with IC50 values of response to diosgenin ranging from 14.02 to 66.10 µM, which were slightly lower than those previously reported [25,26,27,70,71].

Interestingly, the CRPC DU145 and PC3 cells, representing more aggressive stages of PCa, were the most sensitive to 20 µM diosgenin, which also reduced the LNCaP viability but with less extension and did not affect the viability of the non-neoplastic PNT1A cells. Studies using lower diosgenin concentrations (5 µM) showed that it reduced the viability of DU145 cells but not of LNCaP [70], confirming the sensibility of CRPC cells to this steroidal saponin.

Reports indicate that some phytosteroids could reduce cancer growth through interaction with GPER [50,72]. Therefore, we analysed diosgenin’s effects in combination with the GPER agonist G1 in controlling prostate cell fate. Although it did not alter the PNT1A cells’ viability, 20 µM diosgenin reduced the viability of PCa cells, an effect that was much higher in the presence of G1. Moreover, diosgenin, when combined with G1, increased PNT1A viability. Curiously, exposure to diosgenin augmented the expression of GPER in both non-neoplastic and neoplastic cells (not significantly in PC3), which may explain the enhanced results of diosgenin plus G1 in suppressing cell viability compared with the isolated compounds. Other phytosteroids, such as genistein, were found to regulate classical ERs and GPER, up- or downregulating its expression depending on the tissue [72,73,74,75,76]. However, to the best of our knowledge, this is the first report of diosgenin controlling GPER expression.

The changes in prostate cell viability caused by diosgenin treatment were underpinned by the altered expression of cell cycle and cell survival regulators. The oncoprotein c-Myc is a strong promoter of cell cycle progression and cell proliferation [77]. It is also known that the stability of c-Myc is regulated by phosphorylation at the NH2-terminal. ERK-mediated phosphorylation at Ser 62 stabilises c-Myc, whereas glycogen synthase kinase-3-mediated phosphorylation at Thr 58 accelerates ubiquitin-mediated degradation of the oncoprotein [78,79,80,81]. We found that diosgenin decreased the expression of phosphorylated c-Myc in PNT1A cells but increased it in LNCaP and DU145, which suggests that different mechanisms are being activated, likely reflecting the differences observed in cell viability response.

c-Myc is also known to suppress the expression of other cell cycle regulators, namely p21, a cell cycle inhibitor that suppresses the activity of most cyclin-dependent kinases, and tumour suppressor p53 [82,83,84,85,86]. The expression levels of p53 were augmented in the PNT1A and LNCaP cells, and all PCa cell lines showed an increased expression of p21 in response to diosgenin treatment. These results are in agreement with the reduced viability found in PCa cell lines upon exposure to this phytosteroid. Moreover, they may sustain the hypothesis that the increased phosphorylated-c-Myc is being signalled for degradation and not repressing p21 expression [87,88,89]. The WB results have their limitations, and further investigation is needed to corroborate this hypothesis. Nevertheless, in other cancer cell types, it has been shown that diosgenin could revert the TNF effects by increasing the p21 levels [90].

**Figure 8:**
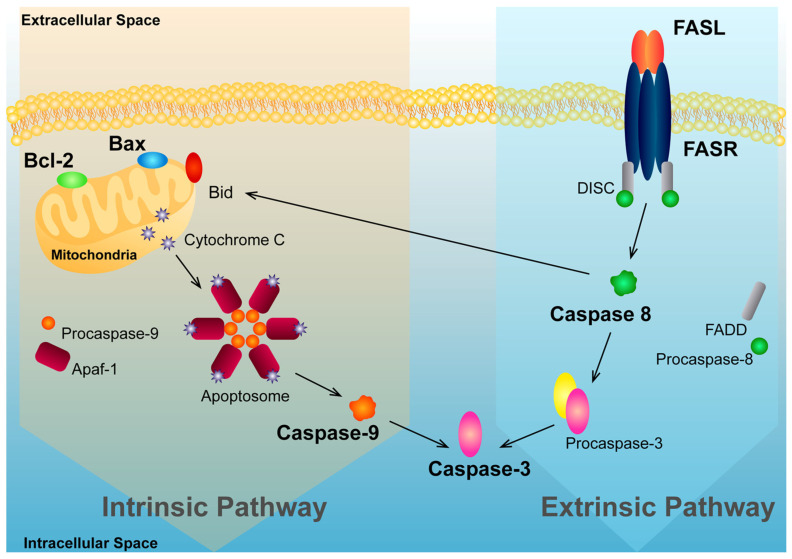
Intrinsic and extrinsic apoptosis pathways converging at the activation of caspase-3. When the intrinsic pathway is activated, Bax promotes the release of cytochrome c from mitochondria, which binds to the apoptotic protease activating factor-1 (Apaf-1) and procaspase-9, cleaving it and originating active caspase-9. It in turn cleaves and activates procaspase-3 into the effector caspase-3. The extrinsic pathway of apoptosis is activated by the interaction between FASR and FASL, resulting in the formation of the death-inducing signalling complex (DISC), which contains FADD and procaspase-8. Upon recruitment by FADD, procaspase-8 is activated by self-cleavage, and then activates other downstream caspases, namely caspase-3, the endpoint of both intrinsic and extrinsic pathways. Furthermore, caspase-8 can cleave (Bcl-2 interacting protein (BID)), which translocates to the mitochondria, triggering cytochrome C release, and following the intrinsic pathway.

We also found that diosgenin treatment augmented the activity of caspase-3, an endpoint of apoptotic signalling [91] (Figure 8), in the PNT1A, LNCaP, and PC3 cells. Moreover, this effect was enhanced in the presence of G1, which suggests an interplay between diosgenin and the actions of GPER. As discussed above, this response could be related to the increase in GPER levels upon diosgenin treatment, which may explain the enhanced effects when both compounds are combined. Furthermore, diosgenin-induced apoptosis of the PC3 cells, not with a significant increase in GPER expression, which was abrogated by GPER knockdown, indicating that the actions of diosgenin driving apoptosis may require GPER. Although other studies have described the proapoptotic effects of diosgenin [26,71,92] and identified the involvement of GPER in phytosteroid actions triggering apoptosis [50,93], this is the first report linking diosgenin with GPER activation in PCa.

Altered caspase-3 activity was followed by changes in the expression of apoptosis regulators. Despite the increase incaspase-3 activity, the Bax/Bcl-2 ratio was reduced by diosgenin in the LNCaP and PC3 cells, which can be explained by diosgenin slightly increasing Bcl-2 expression in the LNCaP cells, but without statistical significance in the PC3-treated cells. The pro-apoptotic/antiapoptotic Bax/Bcl-2 ratio was not determined in the DU145 cells, which do not express the Bax protein [62,63,64], and therefore remained unchanged in the PNT1A-treated cells despite the reduced Bax expression.

The PNT1A cells also displayed reduced caspase-9 expression while showing an increased expression of FASR and caspase-8, mediators of the extrinsic apoptosis pathway [94]. FASR, FASL, and caspase-8 protein expression were also increased in the LNCaP and PC3-stimulated cells, which supports that diosgenin may induce apoptosis by activating the extrinsic pathway.

In the case of the DU145 cells, it cannot be excluded that both the intrinsic and extrinsic apoptotic pathways are activated in response to diosgenin treatment, as these cells displayed increased caspase-9 expression, but also FASL and caspase-8. Another possibility is that the augmentation observed in caspase-9 may be explained by cross activation of the intrinsic pathway through caspase-8 [95], which is reasonable to consider as diosgenin has been shown to inhibit FLIP and activate caspase-8 in the FAS-related apoptotic pathway in human thyrocytes [96].

A well-known characteristic of cancer cells is their metabolic reprogramming, using glycolysis instead of oxidative phosphorylation, even in aerobic conditions [65,66]. Concerning PCa, this switch is mainly associated with the advanced stages of the disease, with the metabolism of primary tumours being less glycolytic and dependent on oxidative phosphorylation [29,67,68,97,98]. Since these metabolic alterations sustain cancer cell growth and progression, being established as a cancer hallmark [29,30], we investigated the effect of diosgenin in modulating the glycolytic profile of prostate cells. This steroid sapogenin enhanced glucose consumption in the PNT1A, DU145 and PC3 cells. Additionally, in LNCaP, diosgenin slightly increased the glucose consumption, although without significance. These results are in line with the augmented expression of GLUTs. In fact, the distinct GLUT isoforms were differentially regulated by diosgenin depending on the cell line, suggesting that each cell line may use different transporters for glucose uptake.

In the glycolytic pathway, glucose is converted into pyruvate, the final product of glycolysis [69]. The PFK1 enzyme catalyses the conversion of fructose 6-phosphate into fructose 1,6-bisphosphate, in a limiting step of glycolysis, indicating the rate of glycolytic flux [69]. Curiously, this enzyme was only augmented in the LNCaP cells and was reduced by diosgenin treatment in the PNT1A and PC3 cells, with no changes observed in the DU145 cells. These results suggest that in the LNCaP cells, glucose enters the glycolytic pathway, but there is no conversion of the resultant pyruvate to lactate. The pyruvate produced in glycolysis can be used in other pathways, namely entering the mitochondria to be oxidised to acetyl CoA and combined with oxaloacetate to start the tricarboxylic acid (TCA) cycle and following oxidative phosphorylation [99]. Our results in the PNT1A and PC3 cells suggest that glucose was not being driven for glycolysis. Instead of entering the glycolytic cascade, glucose can be converted into glycogen through the enzyme GS [100], which was increased in all of the diosgenin-treated cell lines, suggesting that glucose is being stored. Nevertheless, the increased glucose consumption was accompanied by augmented lactate production in the CRPC cells, which was supported by the augmented expression levels of LDH, the enzyme responsible for the conversion of pyruvate into lactate [101] in PC3 cells, and the increased expression of MCT4, the transporter responsible for lactate export to the extracellular space [101,102] in DU145. In CRPC, the increased lactate production was not followed by changes in the expression of PFK and LDH in the case of DU145. Moreover, in the PC3 cells, PFK1 and MCT4 were shown to be downregulated by diosgenin. Thus, these results also suggest that lactate could be produced from other sources, not from glucose [103,104], in CRPC cells. Possible alternative sources of pyruvate include malate transformation by cytosolic malic enzyme or amino acids like alanine, serine, threonine, glycine, cysteine, and tryptophan [105]. Previous research found the ability of diosgenin to reduce the overall glycolytic flux and oxygen consumption rate, inhibiting the mitochondrial oxidative phosphorylation capacity and suppressing the non-mitochondrial respiration of colorectal cancer cells [106]. In contrast to our results, this study also found that diosgenin inhibited glucose uptake and lactate production, with a reduction in *GLUT3*, *GLUT4*, and *pyruvate carboxylase* gene expression and without alterations in the *GLUT1*, *GLUT2*, and *phosphoglycerate kinase* genes [106]. Overall, our results suggest that diosgenin modulates glucose metabolism, mainly driving this metabolite to storage.

Interestingly, the combined treatment of diosgenin plus G1 enhanced the effects of glycolytic metabolism compared to that of the compounds alone, which aligns with the actions of diosgenin in increasing GPER expression and its expected capability of binding and activating the receptor.

## 4. Materials and Methods

### 4.1. Cell Culture and Treatments

Human prostate cell lines PNT1A, LNCaP, DU145, and PC3 were purchased from the European Collection of Cell Cultures (ECACC, Salisbury, UK). PNT1A is a non-neoplastic epithelial cell line, and LNCaP, DU145, and PC3 are neoplastic cell lines representing the androgen-sensitive (LNCaP) and castrate-resistant (DU145 and PC3) metastatic stages of PCa [107,108,109,110].

PNT1A, LNCaP, DU145, and PC3 cells, always with low passages, were maintained in RPMI 1640 medium (Sigma-Aldrich, St. Louis, MO, USA) supplemented with 10% foetal bovine serum (FBS) (Sigma-Aldrich) and 1% penicillin/streptomycin (Sigma-Aldrich) at 37 °C in an atmosphere equilibrated with 5% CO_2_. At 60% confluence, the culture medium was replaced by phenol red-free RPMI 1640 medium (Sigma-Aldrich) containing 5% charcoal-stripped FBS (CS-FBS) (Sigma-Aldrich), and the cells were maintained for an additional 24 h. Then, the cells were treated with the vehicle or diosgenin (purity: 95%, Sigma-Aldrich) alone or in combination with 1 µM of GPER agonist G1 (Tocris Bioscience, Madrid, Spain) for 24 h. This GPER concentration has previously been used by our research group and others with proven results [45].

### 4.2. Cell Viability Assay

The PNT1A (10,000 cells/well), LNCaP (15,000 cells/well), DU145 (5000 cells/well), and PC3 (5000 cells/well) cells were seeded in 96-well plates and treated with diosgenin (0, 0.001, 0.01, 0.1, 1, 10, and 100 µM) for 24 h. Cell viability was determined by the colorimetric 3-(4,5-dimethylthiazolyl-2)-2,5-diphenyltetrazolium bromide (MTT, Sigma-Aldrich) assay. After diosgenin and/or G1 treatment, MTT was added to the cell culture medium (0.5 mg/mL), and the plates were maintained in the dark for 4 h at 37 °C. After incubation, the medium with MTT was removed, and the formed formazan crystals were dissolved in 100 µL DMSO (Honeywell, Charlotte, NC, USA). The absorbance of the resultant purple-coloured solution was measured at 570 nm using the xMark™ Microplate Absorbance Spectrophotometer (Bio-Rad, Hercules, CA, USA). The absorbance value was considered directly proportional to the number of viable cells in each experimental group [111].

### 4.3. Cell Number Analysis

Cell nuclei were stained with Hoechst 33342 (5 µg/mL, Invitrogen, Darmstadt, Germany) for 10 min. Lamellae with control and diosgenin-treated (20 µM) PNT1A, LNCaP, DU145, and PC3 cells were visualised under 400x magnification. Images were acquired using a Zeiss LSM 710 laser scanning confocal microscope (Carl Zeiss, Göttingen, Germany).

### 4.4. Protein Extraction

Cells were homogenised in the appropriate volume of radioimmunoprecipitation assay buffer (RIPA) (150 mM NaCl, 1% Nonidet-P40 substitute, 0.5% Na-deoxycholate, 0.1% SDS, 50 mM Tris, 1 mM EDTA) supplemented with 1% protease inhibitor cocktail and 10% PMSF, kept on ice for 20 min with occasional mixing, and the total proteins were recovered after a 14,000-rpm centrifugation for 20 min at 4 °C. Protein concentration was determined using the BCA protein assay (Thermo Fisher, Waltham, MA, USA) according to the manufacturer’s instructions.

### 4.5. Western Blot

Total proteins (25 µg) were resolved by SDS-PAGE on 12.5% gels and electrotransferred to PVDF membranes (Bio-Rad). After blocking with 5% skimmed dried milk for 1 h, membranes were incubated overnight at 4 °C with the appropriate primary antibodies: rabbit anti-GPER (1:250, ab 39742; Abcam, Cambridge, UK); rabbit anti-phospho-c-Myc (1:1000, #13748, Cell Signalling Technology, Danvers, MA, USA); rabbit anti-p53 (1:1000, FL-393: sc-6243, Santa Cruz Biotechnology, Dallas, TX, USA); rabbit anti-p21 (1:1000, C-19: sc-397, Santa Cruz Biotechnology); rabbit anti-Bax (1:1000, # 2772, Cell Signalling Technology); rabbit anti-Bcl-2 (1:1000, # 2876, Cell Signalling Technology); rabbit anti-caspase-9 p35 (1:1000, H-170:sc-8355, Santa Cruz Biotechnology); mouse anti-caspase-8 p18 (1:100, D-8: sc-5263, Santa Cruz Biotechnology); rabbit anti-FAS receptor (FASR) (1:1000, A-20: sc-1023, Santa Cruz Biotechnology); rabbit anti-FAS ligand (FASL) (1:1000, C-178: sc-6237, Santa Cruz Biotechnology); rabbit anti-GLUT1 (1:1000, CBL 242, Millipore, Darmstadt, Germany); rabbit anti-GLUT2 (1:1000, H-67: sc-9117, Santa Cruz Biotechnology); rabbit anti-GLUT3 (1:1000, H-50: sc-30107, Santa Cruz Biotechnology); rabbit anti-PFK1 (1:1000, H-55: sc-67028, Santa Cruz Biotechnology); rabbit anti-LDH [EP1566Y] (1:10,000, ab 52488, Abcam); rabbit anti-MCT4 (1:1000, H-90: sc-50329, Santa Cruz Biotechnology); mouse anti-GS2 (1:200, G-8: sc-390391, Santa Cruz Biotechnology).

Goat anti-rabbit IgG-HRP (1:40,000, sc:2004; Santa Cruz Biotechnology) or goat anti-mouse IgG-HRP (1:40,000, sc:2005; Santa Cruz Biotechnology) was used as the secondary antibody. A mouse anti-α-tubulin monoclonal antibody (1:10,000, Sigma-Aldrich) or a mouse anti-β-actin monoclonal antibody (1:10,000, Sigma-Aldrich) was used for protein loading control in the WB analyses.

Membranes were incubated with ECL substrate (Bio-Rad) for 5 min and immuno-reactive proteins were visualised with the ChemiDoc™ MP Imaging System (Bio-Rad). Band densities were quantified using the Image Lab software version 3.0.1 (Bio-Rad) and normalised with the respective α-tubulin or β-actin band density.

### 4.6. Caspase-3-like Activity Assay

The caspase-3-like activity was determined spectrophotometrically by detecting the presence of the yellow product p-nitro-aniline (pNA), upon cleavage of the caspase-3 substrate (Ac-DEVD-pNA). In a 96-well plate, 5 µL of the total protein extracts from the PNT1A, LNCaP, DU145, and PC3 cells or PC3 si-RNA transfected cells were incubated overnight at 37 °C with 85 µL of assay buffer (20 mM HEPES, pH 7.4, 2 mM EDTA, 0.1% CHAPS, 5 mM DTT) and 200 µM of Ac-DEVD-pNA (Sigma-Aldrich). The yellow colour obtained by the release of pNA was measured at 405 nm using an xMark™ Microplate Absorbance Spectrophotometer (Bio-Rad). The amount of generated pNA was quantified by extrapolation with a standard curve. All measurements were normalised to the total amount (µg) of protein in each sample.

### 4.7. GPER Gene Knockdown

PC3 (100,000 cells/well) was seeded in 6-well plates containing 2 mL/well of RPMI 1640 medium (Sigma-Aldrich) supplemented with 10% foetal bovine serum (FBS) (Sigma-Aldrich) and 1% penicillin/streptomycin (Sigma-Aldrich). After 24 h at 37 °C in an atmosphere equilibrated with 5% CO_2_, the cell culture medium was replaced by phenol red and antibiotic-free medium. At 50% confluence, PC3 cells were transfected with 40 nM of a siRNA targeting GPER (GPER1, Silencer Select, s6053, Ambion, Carlsbad, CA, USA), or scramble siRNA (Negative Control, Silencer Select, 4390846, Ambion) for 24 h. For this purpose, the appropriate quantity of si-scramble and si-GPER (per well) were diluted in 150 μL of Opti-MEM^®^ (mix A), and simultaneously, 9 uL of lipofectamine RNAiMAX (Invitrogen, Carlsbad, CA, USA) was diluted in 150 μL of Opti-MEM^®^ (mix B). Mixes A and B were incubated for 5 min at room temperature (RT) and then combined, allowing for the formation of siRNA:lipofectamine RNAiMAX complexes for an additional 20 min at RT. Then, the siRNA:lipofectamine RNAiMAX complexes were added to each well and the cells were incubated at 37 °C in a humid atmosphere equilibrated with 5% CO_2_ for an additional 24 h. *GPER* gene knockdown for 24 h transfection was evaluated by WB analysis.

### 4.8. Quantification of Glucose and Lactate

The concentration of glucose and lactate in the culture medium of the control and diosgenin and/or G1-treated PNT1A, LNCaP, DU145, and PC3 cells was determined by means of spectrophotometric analysis using commercial kits (Spinreact, Girona, Spain) following the manufacturer’s instructions. For glucose and lactate quantification, 1 µL of cell culture medium of each sample was placed in a 96-well plate and mixed with 100 µL of kit reaction buffer. The plate was incubated at 37 °C for 10 min, and the absorbance was measured at 505 nm (xMark™ Spectrophotometer, Bio-Rad). Glucose consumption and lactate production after 24 h of treatment were calculated relative to the initial concentration of these metabolites at 0 h of stimulation. All measurements were normalised to the cell number in each sample.

### 4.9. Lactate Dehydrogenase Enzymatic Activity Assay

LDH activity was measured using a commercial kit (Spinreact) following the manufacturer’s instructions. One microlitre of cell protein extract was added to 150 µL of a kit reagent in a 96-well plate and incubated for 1 min at 37 °C. The initial absorbance was acquired, followed by subsequent readings every min for 3 min. LDH catalyses the reduction of pyruvate to lactate with the oxidation of NADH to NAD+. Thus, the activity of LDH in the sample is proportional to the rate of decrease in NADH concentration, which was measured spectrophotometrically at 340 nm using an xMark™ Microplate Absorbance Spectrophotometer (Bio-Rad). LDH activity was calculated by normalising the values to the total protein concentration in each sample (U/L/µg of protein).

### 4.10. Statistical Analysis

Statistical analysis was performed using GraphPad Prism v6.01 (GraphPad Software, Inc., La Jolla, CA, USA). The statistical significance of differences between the experimental groups was evaluated by the unpaired *t*-test with the appropriate correction or one-way ANOVA, followed by the adequate post-test. *p* < 0.05 was considered statistically significant. All experimental data are shown as the mean ± standard error of the mean (S.E.M).

## 5. Conclusions

In conclusion, our results showed that diosgenin diminished cell viability, increased apoptosis, and modulated the glycolytic metabolism of PCa cells, supporting its antitumorigenic role in PCa. Moreover, we showed that the actions of diosgenin are potentiated in the presence of G1 and require GPER-mediated signalling. The present findings also underlie the potential therapeutic interest of diosgenin (alone or in combination with G1), namely targeting the more aggressive stages of PCa.

## Data Availability

The data presented in this study are available in this article.
